# Gctf: Real-time CTF determination and correction

**DOI:** 10.1016/j.jsb.2015.11.003

**Published:** 2016-01

**Authors:** Kai Zhang

**Affiliations:** Medical Research Council Laboratory of Molecular Biology, Division of Structural Studies, Francis Crick Avenue, Cambridge CB2 0QH, UK

**Keywords:** 1D (2D, 3D), one(two, three) dimensional, cryoEM, cryo-electron microscopy, CCC, cross-correlation coefficient, CCD, Charge Coupled Device, CCF, cross-correlation function, CTF, contrast transfer function, DQE, detective quantum efficiency, EPA, equiphase averaging, FFT, Fast Fourier Transform, GPU, Graphic Processing Unit, HAV, hepatitis A virus, LAS, logarithmic amplitude spectra, NFS, Network File System, SNR, signal to noise ratio, SSD, Solid State Disk, Contrast transfer function, Cryo-electron microscopy, GPU program, CTF determination

## Abstract

Accurate estimation of the contrast transfer function (CTF) is critical for a near-atomic resolution cryo electron microscopy (cryoEM) reconstruction. Here, a GPU-accelerated computer program, Gctf, for accurate and robust, real-time CTF determination is presented. The main target of Gctf is to maximize the cross-correlation of a simulated CTF with the logarithmic amplitude spectra (LAS) of observed micrographs after background subtraction. Novel approaches in Gctf improve both speed and accuracy. In addition to GPU acceleration (e.g. 10–50×), a fast ‘1-dimensional search plus 2-dimensional refinement (1S2R)’ procedure further speeds up Gctf. Based on the global CTF determination, the local defocus for each particle and for single frames of movies is accurately refined, which improves CTF parameters of all particles for subsequent image processing. Novel diagnosis method using equiphase averaging (EPA) and self-consistency verification procedures have also been implemented in the program for practical use, especially for aims of near-atomic reconstruction. Gctf is an independent program and the outputs can be easily imported into other cryoEM software such as Relion (Scheres, 2012) and Frealign (Grigorieff, 2007). The results from several representative datasets are shown and discussed in this paper.

## 1 Introduction

Recent progress has allowed cryo-electron microscopy (cryoEM) to determine structures of bio-macromolecules to near-atomic resolution (Nogales and Scheres, 2015). This is due to developments in multiple fields, but especially better detectors and image processing methods (Bai et al., 2015). The significantly improved detective quantum efficiency (DQE) of direct detectors, such as Falcon II and K2 summit, makes the quality of the cryoEM reconstructions much better than when using traditional CCD or film (Bai et al., 2015). Recording movies on these detectors allows motion correction of entire micrographs or individual particles, which makes critical improvements for high resolution reconstruction. More and more structures at near-atomic or atomic resolution are being solved recently by cryoEM (Amunts et al., 2015, Bartesaghi et al., 2015, Jiang et al., 2015, Paulsen et al., 2015, Taylor et al., 2015, Urnavicius et al., 2015, Zhao et al., 2015).

In contrast to a simple projection of a 3-dimensional object, the cryoEM image of vitrified specimen is modulated by contrast transfer function (CTF). Because of the thin vitreous ice film, the image formation can be well described by weak-phase approximation (Wade, 1992). Based on this approximation, the phase contrast is dominant while the amplitude contrast is very small. Therefore, the major factors that affect the CTF of cryoEM image formation are the defocus and aberration of lens. The effect of these factors makes CTF a frequency-dependent oscillatory function, modulating both the amplitudes and phases of the image. Original information of the images must be corrected using accurate CTF parameters in order to obtain a reliable 3D reconstruction. The oscillation of the CTF becomes more severe at higher frequency or under a higher defocus. For this reason, information restoration from cryoEM image is quite challenging, especially at high frequency, which makes accurate CTF determination an important factor for near-atomic 3D reconstructions.

There are currently several programs available for CTF determination (Ludtke et al., 1999, Mallick et al., 2005, Mindell and Grigorieff, 2003, Penczek et al., 2014, Shaikh et al., 2008, Sorzano et al., 2004, Tang et al., 2007, Vargas et al., 2013, Voortman et al., 2011). In a recent work, researchers systematically studied the performance of different programs (Marabini et al., 2015). Each of the programs has its own advantages for certain purposes. The popular program CTFFIND3 (Mindell and Grigorieff, 2003) shows the most self-consistent results using real datasets in this benchmark test, in spite of a slightly lower rank using simulated micrographs. However, with the fast development of cryoEM, a lot of new challenges are being required for daily image processing. One challenging requirement is to further improve CTF accuracy for 3D reconstruction at near-atomic or real atomic resolution. Higher speed without sacrificing the accuracy is also helpful to facilitate data processing with the development of automatic data collection at higher throughput. Besides, automatic self-consistency verification of the CTF determination and quality evaluation of the micrographs will greatly facilitate the ultimate goal of automation in cryoEM.

Here a robust GPU-accelerated computer program called Gctf for CTF determination, refinement and correction is presented. GPU acceleration as well as an optimized programming strategy makes Gctf very fast. It can easily process thousands of micrographs within minutes using a single GPU card. The accuracy of the global CTF determination was verified by both manual scrutiny and automatic verification. Astigmatism-based rotational averaging or what is called equiphase averaging (EPA) in Gctf makes the visibility of Thon rings significantly improved for better diagnosis. Gctf was tested using a variety of parameters, showing stable ranges of parameter selection and thus its potential power for CTF automation of many types of micrographs. Micrographs from many datasets collected at the MRC-LMB (Cambridge) and several other collaborating institutes proved its accuracy, speed, convenience and robustness in practical use. In almost all cases, there was no need for parameter optimization. Gctf also performed well with a number of deliberately selected challenging micrographs.

Local refinement and movie processing have also been implemented in Gctf. Local defocus refinement for each single particle makes significant improvements for 3D reconstructions carried out with datasets that have large defocus variation. Refinement of defocus of each frame in a movie provides a way of tracking frame movement in the *Z*-direction during imaging. Beside the determination and refinement of defocus in Gctf, CTF correction, automatic self-consistency verification and micrographs quality evaluation are also available for better automation of cryoEM data processing.

## 2 Theory and methods

### 2.1 Application, usage and typical input/output of Gctf

Gctf is basically designed to estimate the unknown CTF parameters of EM micrographs. A summary of capability in the current version (1.0) of Gctf is listed as follows:

(a) Determine overall CTF parameters of
  (a.1) each micrograph (basic application);
  (a.2) each particle stack from the same micrograph;
  (a.3) each movie stack by
    (a.3.1) coherent averaging;
    (a.3.2) incoherent averaging;
(b) Refine user-provided CTF parameters of (a.1), (a.2), (a.3);
(c) Refine local CTF parameters based on (a) for
  (c.1) each frames from the same movie;
  (c.2) each particle
    (c.2.1) using coordinate files as input;
    (c.2.2) using particle stacks as input;
    (c.2.3) from auto-detection by
      (c.2.3.1) user provided templates
      (c.2.3.2) Gaussian convolution
(d) Auto-check CTF determination results after (a), (b) or (c) by
  (d.1) self-consistency verification;
  (d.2) evaluation of the micrograph quality;
  (d.3) overall defocus or astigmatism variation;
  (d.4) local defocus variation;
(e) Generate better diagnostic file after (a), (b) or (c) by EPA
(f) Perform CTF correction after (a), (b) or (c).

Additional details are summarized in Table 1 of (Zhang, 2015). All these kinds of processing only require a single and simple command, running in batch mode for the entire dataset:

Gctf[options]<micrographs>

Only MRC format are supported in the current version of Gctf. There are ∼40 parameters or options as inputs. The pixel size, spherical aberration, high tension, amplitude contrast are regarded as the most basic parameters and should be specified if they are not the same as default. All other parameters are only suggested for challenging cases or advanced usage (e.g. local or movie refinement).

The basic output of Gctf contains four parts: (1) the standard output which gives real-time information about the processing of the micrographs; (2) the log files which contain all the necessary input and output parameters for each micrograph; (3) the diagnosis file in MRC format; (4) a STAR file which contains all the determined CTF parameters. Local or movie refinement outputs additional files. The program is fully compatible with Relion (Scheres, 2012) and the log files and output STAR files can be directly used for further 2D or 3D classification. The STAR files can either contain CTF parameters for entire micrographs or individual particles.

CTF parameters from the text log file can also be easily extracted and used for other programs such as Frealign (Grigorieff, 2007), EMAN (Ludtke et al., 1999, Tang et al., 2007), Spider (Shaikh et al., 2008) and Xmipp (Sorzano et al., 2004) etc. The diagnosis file is similar to that of that of CTFFIND3 (Grigorieff, 2007, Mindell and Grigorieff, 2003) but contains additional user defined options.

Details will be discussed in the following sections: the basic knowledge of CTF (Section 2.2), a defocus inaccuracy criterion widely used in Gctf (Section 2.3), the main target of Gctf (Section 2.4) and its implementation (Section 2.5), local (Section 2.6) and movie (Section 2.7) refinement for better CTF accuracy, resolution-extension and Bfactor-switch methods for more challenging case (Section 2.8), equiphase averaging for better diagnosis (Section 2.9), self-consistency verification and micrograph evaluation (Section 2.10), the acceleration by GPU and improved algorithm and strategy (Section 2.11).

### 2.2 Definition of contrast transfer function

Image formation in a weak-phase approximation is modulated by the CTF which can be defined as Eq. (1).

(1) $$\text{CTF}\;(\overset{\to }{s})=-\sqrt{1-A^{2}}\cdot \sin(\gamma (\overset{\to }{s}))-A\cdot \cos(\gamma (\overset{\to }{s}))=-\sin(\Delta \phi +\gamma (\overset{\to }{s}))$$

where $\overset{\to }{s}$ is the spatial frequency; A is the amplitude contrast coefficient; $\gamma (\overset{\to }{s})$ is a function of $\overset{\to }{s}$ representing the varying phases of the CTF, while Δϕ is a global phase shift contributed by amplitude contrast using empirical values.

Ideally an image can be regarded as a projection of a 3D object convoluted by the CTF. In other words, the Fourier transform of an ideal image is the Fourier transform of a projection multiplied by the CTF. Note that an envelope function and noise severely affect the real image formation which must be taken into consideration for reliable CTF determination and correction.

Defocus and spherical aberration of the microscope lens are the two major factors that affect the values of $\gamma (\overset{\to }{s})$ formulated as Eq. (2). The effect by other factors such as coma aberration is ignored in the current CTF determination method.

(2) $$\gamma (\overset{\to }{s})=\gamma (s\text{,}\theta )=-\frac{\pi }{2}C_{s}\lambda^{3}s^{4}+\pi \lambda z(\theta )s^{2}$$

where s is the modulus of $\overset{\to }{s}$, $s=|\overset{\to }{s}|$ and $\overset{\to }{s}=s\cdot e^{i\theta }$; λ is the wavelength of an electron; $C_{s}$ is the spherical aberration coefficient; z(θ) is the defocus in the direction with an varying azimuthal angle *θ*, which can be precisely calculated using the following function Eq. (3).

(3) $$z(\theta )=z_{u}\cos^{2}(\theta -\theta_{\mathrm{ast}})+z_{v}\sin^{2}(\theta -\theta_{\mathrm{ast}})$$

where the defocus z(θ) is determined based the three parameters $\left(z_{u}\text{,}z_{v}\text{,}\theta_{\mathrm{ast}}\right)$, $z_{u}$ and $z_{v}$ represents the maximum or minimum defocus; $\theta_{\mathrm{ast}}$ is the fixed angle between axis $z_{u}$ and *x*-axis of Cartesian coordinate system. All the parameters, including the rest parts of this paper, follow the convention as proposed previously (Heymann et al., 2005).

### 2.3 Defocus inaccuracy related phase error criterion

The accuracy of defocus determination is very important for high-resolution cryoEM reconstructions. Assuming the difference between the true defocus of a micrograph and the estimated defocus is Δz, the phase error Δγ(s) is calculated by Eq. (4):

(4) $$\Delta \gamma (s)=\pi \lambda \Delta {\mathrm{zs}}^{2}$$

Derived from Eq. (4), the defocus-inaccuracy dependent phase error is proportional to frequency squared for a certain micrograph Eq. (5).

(5) $$\frac{\Delta \gamma (s_{1})}{\Delta \gamma (s_{2})}=\frac{s_{1}^{2}}{s_{2}^{2}}$$

**Fig. 1:**
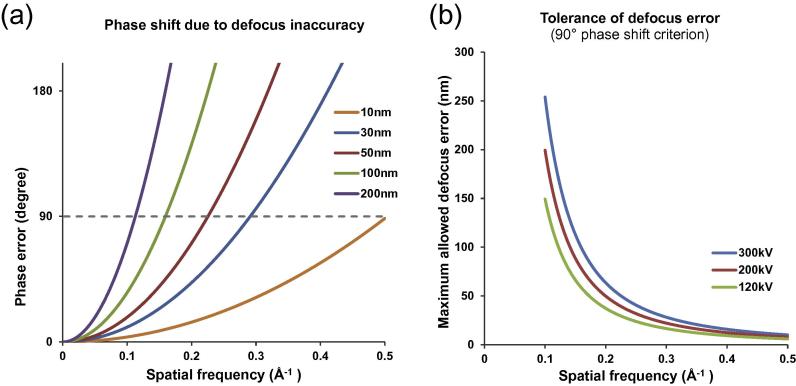
Relationship between CTF phase error and defocus inaccuracy. (a) The errors of CTF phases by different levels of defocus inaccuracy at 300 kV high tension. The dashed gray line represents the threshold for 90° phase shift criterion. (b) Based on the 90° criterion from (a), the maximum defocus inaccuracy allowed at various resolutions for three typical high tension values (300 kV, 200 kV and 100 kV) are plotted.

Obviously from Eqs. (4), (5), an error in CTF determination, which can be ignored for a lower resolution reconstruction, might cause a critical error at high resolution. If the CTF is not properly determined, there are increasing phase errors against the frequency. The contrast of CTF is inverted for a 180 degree phase error. When this error is smaller than 90 degree, the probability to have the correct contrast of CTF is more than 50%. Gctf uses such a 90 degree criterion in order to guarantee at least half of information from the EM images after CTF correction. Based on this criterion, CTF phase error versus frequency for different defocus errors between 10 nm and 200 nm were plotted (Fig. 1a). The maximum allowed CTF defocus errors were plotted against frequency for three typical voltages used in cryoEM reconstruction s (Fig. 1b).

In practice, defocus inaccuracy is only one of the factors that cause CTF phase error. Magnification distortion, chromatic or comatic aberration (Glaeser et al., 2011), astigmatism inaccuracy, mechanical and beam induced movement of the samples, curvature or deformation of the carbon substrate (Shatsky et al., 2014), sample thickness (DeRosier, 2000) can all contribute to the phase error during an experiment. Data processing can also lead to large phase errors, especially at high frequency. Although Gctf uses this 90 degree criterion, it should be noted that the highest quality micrographs might need a stricter criterion in practice.

### 2.4 CTF determination target

The basic target is to estimate three unknown parameters $\left(z_{u}\text{,}z_{v}\text{,}\theta_{\mathrm{ast}}\right)$ as described in Eq. (6):

(6) z^(zu,zv,θast)=argzmaxCCLn|F(s→)|-Bg(Ln|F(s→)|),|CTFsim(s→)|·e-B4s2

where z^(zu,zv,θast) is the estimated CTF parameters; $\left|F(\overset{\to }{s})\right|$ is the amplitude spectrum; Bg is the estimated background from $\mathrm{Ln}|F(\overset{\to }{s})|$, the logarithmic amplitude spectra (LAS); ${\text{CTF}}_{\mathrm{sim}}(\overset{\to }{s})$ is the simulated CTF; CC represents the cross-correlation; B is an input B-factor used to down-weight high-frequency.

### 2.5 Flow-chart of Gctf

**Fig. 2:**
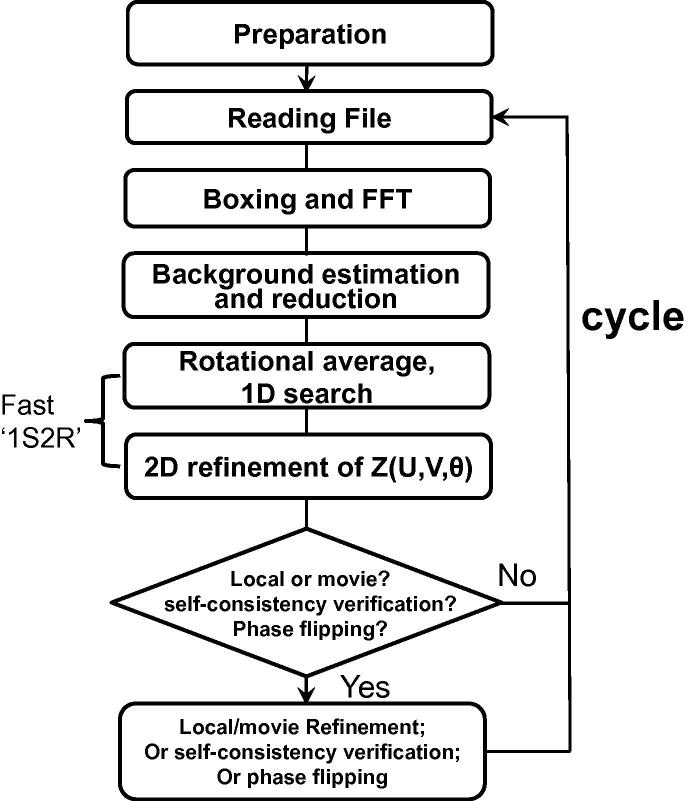
Flow chart of Gctf.

The overall flow chart of Gctf can be described as shown in Fig. 2. The preparation step contains the following process: handling input/output parameters; setting up the program running environment (e.g. checking and assigning the GPU device); allocating necessary memory for both CPU and GPU; pre-calculating sharable parameters and data.

**Fig. 3:**
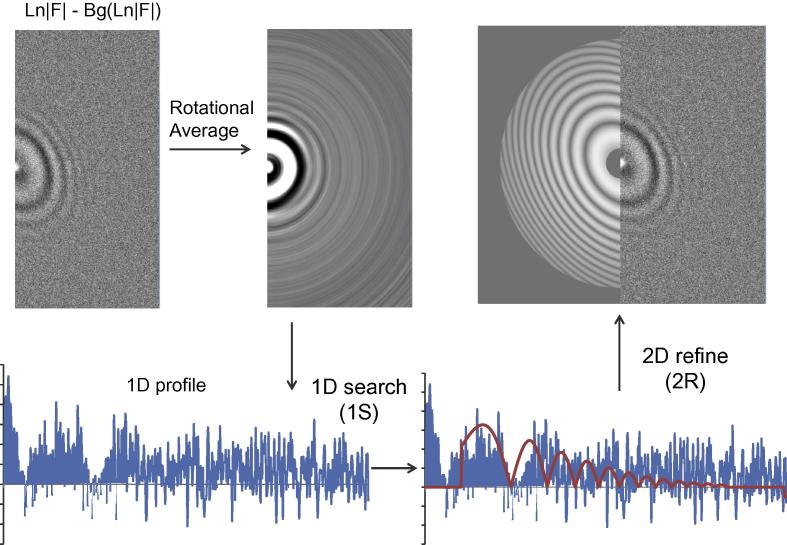
Flow chart of Gctf using a real micrograph. A micrograph with significant astigmatism is presented to demonstrate the procedure clearly.

The CTF determination contains the following steps (Fig. 2): read and write files; box out sub-areas, perform a series of FFT to generate an averaged amplitude spectra; convert the averaged amplitude spectra to LAS; estimate and subtract the background from LAS; circularly average LAS to get a 1-dimensional (1D) profile (Fig. 3a and b); search for the average defocus that best fits the observed 1D profile; perform a 2-dimensional (2D) refinement of all three parameters $\left(z_{u}\text{,}z_{v}\text{,}\theta_{\mathrm{ast}}\right)$. The key procedure of ‘1D search plus 2D refinement’ is called ‘1S2R’ briefly in the rest parts of this paper. In addition, local and movie refinement, self-consistency verification or phase flipping can be performed if specified. Gctf then reads and processes another file.

The estimation of the background uses a box-convolution of $\mathrm{Ln}|F(\overset{\to }{s})|$, similar to CTFFIND3 (Mindell and Grigorieff, 2003) but different. In contrast to CTFFIND3 which uses the square root of power spectra $\sqrt{\left|P(\overset{\to }{s})\right|}$ (or $\left|F(\overset{\to }{s})\right|$), Gctf uses $\mathrm{Ln}|F(\overset{\to }{s})|$ to down-weight the strong signals at low frequency that tend to dominate and mislead the CTF determination. Background is estimated in 2D using logarithmic amplitude spectra (LAS), $\mathrm{Ln}|F(\overset{\to }{s})|$. Circular average was performed using the background-subtracted LAS image.

The averaged defocus $z_{a}=(z_{u}+z_{v})/2$ is estimated using the circularly averaged 1D profile (blue curve in Fig. 3c). A large range of defocuses (e.g. 5000–90,000 Å at a step size of 500 Å by default) were used to generate a series of CTF curves in 1D. A cross-correlation function (CCF) is then calculated between these simulated CTF and the observed 1D profile. The estimated defocus is the one (red curve in Fig. 3d) which yields the maximum cross-correlation coefficient (CCC) (the Pearson product-moment, the same for the rest part of this paper).

The 1D result is extended for 2D using the parameter group $\left(z_{a}+\Delta z/2\text{,}\;z_{a}-\Delta z/2\text{,}\;\theta_{R}\right)$ as initial seed for $\left(z_{u}\text{,}\;z_{v}\text{,}\;\theta_{\mathrm{ast}}\right)$. Δz is the input astigmatism as estimation and will be refined in 2D. $\theta_{R}$ is randomly generated in the range of 0–30°. Five more seeds of $\theta_{R}$ are then generated using $\theta_{R}+N*\;30$, where N belongs to {1, 2, 3, 4, 5}. Coarse 2D refinement for each parameter group was performed in parallel. The maximized CCCs from the six seeds were compared and the best one was selected for accurate refinement by Simplex method. Gctf only normalizes the CCC at last step for faster speed.

### 2.6 Local refinement strategy

The accuracy of defocus for near-atomic resolution (<4.0 Å) should be at least better than 40 nm at 300 kV as described (Fig. 1). However, stage tilt, uneven ice, a distorted supporting carbon film or charging can all lead to the defocus variation among particles within a cryoEM micrograph. Simply considering the tilt of micrograph will not generate accurate local defocus caused by nonlinear factors. Therefore, a new local refinement strategy for each particle in one micrograph is implemented in Gctf to solve this problem without assuming any model for defocus variation.

Gctf does a two-step estimation of single particle CTF determination to deal with low signal to noise ratio (SNR) at high frequency. First, it determines the global CTF parameters for an entire micrograph. Using these global values as initial estimation, it does a local refinement for each particle instead of *ab initial* CTF determination. The target is to estimate the amplitude spectra of each particle together with its surrounding areas. It uses Gaussian weighting according to the distances between the centers of the particles as described in Eq. (7).

(7) $$|F_{i_{\mathrm{ave}}}(\overset{\to }{s})|=\frac{\sum_{j=1}^{n}\left(e^{-\frac{d_{\mathrm{ji}}^{2}}{2\delta_{d}^{2}}\cdot \frac{s^{2}}{2\delta_{s}^{2}}}\cdot |F_{j}(\overset{\to }{s})|\right)}{\sum_{j=1}^{n}\left(e^{-\frac{d_{\mathrm{ji}}^{2}}{2\delta_{d}^{2}}\cdot \frac{s^{2}}{2\delta_{s}^{2}}}\right)}$$

where $\left|F_{i_{\mathrm{ave}}}\right|$ is the averaged amplitudes of $i_{\text{th}}$ particle; $\left|F_{j}\right|$ the amplitudes of the $j_{\text{th}}$ neighbor; $d_{\mathrm{ji}}$ is the distance between particle i and its neighbor i (including i itself) and $\delta_{d}$ is the standard deviation of all distances to all neighbors; $\delta_{s}$ is similar to $\delta_{d}$ but with a down-weighting of high-frequency. Note that the combination of the weighting by distance and frequency is a multiplication of the exponent.

There are two different weighted averaging approaches in Gctf for local refinement. One approach simply takes everything in the neighboring areas into account. The other approach uses the coordinates of picked particles or user defined boxes. The coordinates are either provided by the user or auto-detected by cross-correlation with a Gaussian function or templates.

### 2.7 CTF refinement for movies

One of the biggest advances in cryoEM recently is the invention of direct electron detectors which allow movie recording. Beam induced movement correction using movies has greatly improved the resolution of the final reconstruction (Bai et al., 2013, Li et al., 2013). The movement in the *X* or *Y* direction of a micrograph is usually around several Ångstroms (e.g. 1–10 Å), while the *Z*-direction movement can be over a hundred Ångstroms (Russo and Passmore, 2014). Although the movement is dominantly in the *Z*-direction, the small movement in the *XY* plane severely affects the quality of cryoEM micrographs. Motion correction programs normally consider only the drift in the *XY* plane because the eucentric height of the object does not affect its ideal 2D projection. However, EM micrographs are modulated by CTF, which is sensitive to *Z*-height changes. Beam induced movement might change the CTF from frame to frame. A hundred Ångstrom movement is not a significant change even up to a 3 Å reconstruction, but Fig. 1 suggests it might help to improve a reconstruction close to 2 Å.

Accurate defocus refinement for movie frames is implemented in Gctf to deal with large movement in the *Z*-direction. Similar to local defocus refinement, movie defocus refinement is performed in two steps. First, global CTF parameters are determined for the averaged micrograph of motion-corrected movies. Then based on the global values, parameters for each frame are refined using an equally weighted average of adjacent frames (suggested 5–10) to reduce the noise. Two options are provided in Gctf: coherent averaging Eq. (8) or incoherent averaging Eq. (9).

(8) $$|F_{i_{\mathrm{ca}}}(\overset{\to }{s})|=\left|\overset{i+N/2}{\underset{j=i-N/2}{\sum }}\frac{F_{j}(\overset{\to }{s})}{N}\right|$$

(9) $$|F_{i_{\mathrm{ica}}}(\overset{\to }{s})|=\overset{i+N/2}{\underset{j=i-N/2}{\sum }}\frac{\left|F_{j}(\overset{\to }{s})\right|}{N}$$

where $\left|F_{i_{\mathrm{ca}}}(\overset{\to }{s})\right|$ represents the coherent averaging of $i_{\text{th}}$ frame and $i_{\text{th}}$ the incoherent averaging; N is the number of frames to be averaged.

### 2.8 Resolution–extension and Bfactor-switch

Strong structure factors at low spatial frequencies can lead to CTF determination bias. Direct CTF determination at high frequency using the ‘1S2R’ procedure might fail in the case of large astigmatism due to severe oscillation of CTF. Two options are provided to deal with CTF determination at near-atomic resolution for micrographs that have very large astigmatism. They both make the ‘1S2R’ procedure more robust in such challenging case. One option is ‘resolution–extension (RE)’ and the other is ‘Bfactor-switch (BS)’. In the first method, Gctf determines initial CTF parameters using a relatively lower resolution ring (e.g. 50–10 Å by default). These parameters are passed as input to the next step of CTF refinement using a higher range (e.g. 15–4 Å). In the second method, Gctf uses a larger Bfactor (e.g. 500 Å^2^) to significantly down-weight high frequency for initial CTF determination. Then it switches to a smaller Bfactor (e.g. 50 Å^2^) to refine the previously determined CTF parameters. Either method shows its power to deal with some challenging cases (detailed results in Section 3.5). The combination (‘REBS’) can even work slightly better in certain cases.

### 2.9 Equiphase averaging (EPA)

**Fig. 4:**
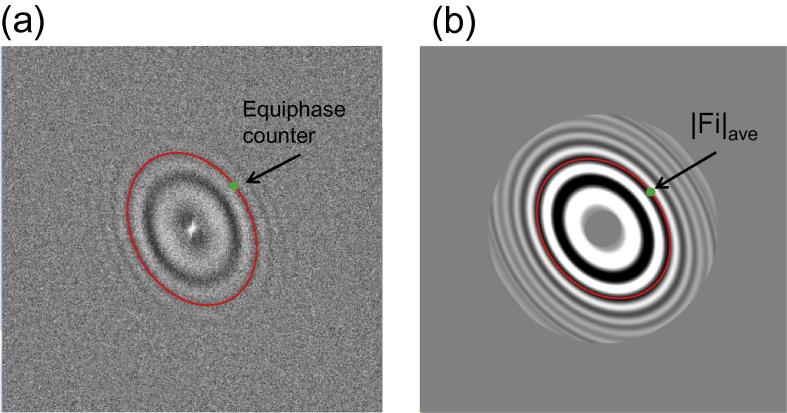
Equiphase average. (a) The logarithmic amplitude spectra (LAS) after background reduction. The green point is the target pixel to be averaged. The red line represents all pixels with equiphases for the green point in this image. (b) A typical equiphase averaged LAS image. Resolution lower than 50 Å or higher than 7 Å has been excluded.

The astigmatism of practical datasets can range from several hundred to over a thousand Ångstroms. One of the tested datasets (hepatitis A virus, HAV) (Wang et al., 2015) had the astigmatism of ∼1800 Å but still reached 3.4 Å resolution. High astigmatism makes the Thon rings in the power spectra approximately elliptical, which means circular averaging, Eq. (10) with constant s(θ), will not provide a good estimation for them. Therefore the EPA approach is proposed after CTF determination for better diagnosis. The idea is to average the amplitudes of the micrograph FFT which have the same CTF phases $\gamma (\overset{\to }{s})$ (Fig. 4 and Eqs. (11), (12)).

(10) $$|F_{\mathrm{ave}}({\overset{\to }{s}}_{0})|=|F_{\mathrm{ave}}(\theta_{0}\text{,}s_{0})|=\frac{1}{\pi }\int_{-\frac{\pi }{2}}^{\frac{\pi }{2}}|F(\theta \text{,}s(\theta ))|d\theta$$

For a specific point with frequency magnitude *s*_0_ and azimuthal angle *θ*_0_ in Fourier space, Eq. (10) represents how the rotational average of the amplitude $\left|F_{\mathrm{ave}}\right|$ is calculated. If the frequency s is independent of θ, or in other words s is always equal to $s_{0}$, it is the circular average. Using the method EPA, Gctf only averages the amplitudes with the same CTF phases as in Eq. (11). For any angle θ, the frequency magnitude s(θ) involved in the average is calculated using Eq. (12).

(11) $$\gamma (s\text{,}\theta )=-\frac{\pi }{2}C_{s}\lambda^{3}s^{4}+\pi \lambda z(\theta )s^{2}=\gamma ({\overset{\to }{s}}_{0})$$

(12) $$s(\theta )=\sqrt{\frac{z(\theta )-\sqrt{z^{2}(\theta )-2C_{s}\lambda \gamma ({\overset{\to }{s}}_{0})/\pi }}{C_{s}\lambda^{2}}}$$

The defocus z(θ) in Eqs. (11), (12) is calculated using the definition in Eq. (3). The correct solution for s(θ) derived from Eq. (11) is the one that lies within the normal range of frequency (smaller than Nyquist). Note that the calculated s(θ) looks elliptical but not ideally elliptical because of the spherical aberration. This is important to generate better Thon rings at near atomic resolution than simply doing elliptical averaging. In the case of Cs-corrected micrograph where Cs is zero or near zero, it will cause problem using Eq. (12) in EPA method. Another solution is described in Eq. (13). This is automatically chosen based on the user input Cs.

(13) $$s(\theta )=\sqrt{\frac{\gamma ({\overset{\to }{s}}_{0})}{\pi \lambda z(\theta )}}$$

### 2.10 Self-consistency verification and micrograph quality evaluation

The CTF determination are affected by both the bias and fitting error. Low resolution bias comes from over fitting of strong false signals in a certain range of frequency, which normally derives from the background (e.g. big ice contamination) or significant structural information (e.g. ring-like structure or ring-like features in the structure). In contrast, random error mainly reflects the quality of the micrograph itself and only affects CTF determination at high frequency. Gctf deals with low resolution bias by independent resolution ring refinement and uses these results to estimate the accuracy and reliability of CTF determination (Fig. 1 in (Zhang, 2015)). Starting with the global values plus deliberately added error (180° phase shift for highest resolution of that ring), Gctf recalculates the CTF parameters for each resolution ring (e.g. 20-8 Å, 15-6 Å, 10-5 Å, 6-4 Å). In good cases, the CTF determination results from new refinement will converge to original values from a wider resolution ring (e.g. 50-4 Å by default), while in bad cases the refinement becomes unstable and eventually generates big differences. Gctf converts the defocus difference to phase shift as defined in Eq. (4) and Fig. 1. A quality score is then defined using this phase shift: 1 (>*π*), 2 (*π*-*π*/2), 3 (*π*/2-*π*/4), 4 (*π*/4-*π*/8), 5 (<*π*/8). It is recommended to use micrographs with quality score equal or higher than 3 which is defined as ‘USABLE’ in Gctf.

Gctf also determines the quality of information at different resolution ring for each micrograph by calculating CCC between the simulated CTF and observed LAS after background subtraction. For each resolution ring, if the CCC is larger than zero in the condition self-consistency verification is convergent, it is regarded as usable. When the CCC values begin to oscillate above and below zero, the resolution ring is assumed not to contain usable information. In Gctf, convergence is prior to CCC evaluation. In other word, if the CTF determination does not converge for a certain micrograph, there is no need for further quality evaluation of this micrograph by CCC. It is important to make sure the CTF determination is convergent before doing anything on the micrograph quality evaluation. There are two reasons: first, if the CTF determination is essentially wrong, the micrograph quality could be evaluated to be very low even if it is good; second, if the CTF determination is biased at high resolution (e.g. one Thon ring off at 4 Å), the CCC is still very high, leading to a wrong judgment. The first problem is easy to fix by manual check. However, the second one is very challenging, because none of the current alternative CTF determination programs could guarantee a perfect fitting at near-atomic resolution due to invisible Thon rings. Gctf provide this self-consistency verification automatically and the powerful EPA for manual check.

The values determined by Gctf can also be automatically checked for self-consistency in real time according to the historical refinement results if this option is specified. One criterion is that the astigmatism (absolute difference between *z_v_* and *z_u_*, e.g. 600 Å) is fixed for a certain dataset when the alignment of microscope keeps stable. Another useful check follows the observation that people tend to collect data at a certain range of defocus for a specific cryoEM sample. Once the difference of defocus value from the average is suddenly much larger (e.g. 3 times) than the standard deviation or if the astigmatism suddenly varies more than expected, the micrograph or the CTF determination is potentially abnormal.

### 2.11 Acceleration by GPU, ‘1S2R’ and optimized programming strategy

Gctf was written in the GPU programming language CUDA (C-language version) (https://developer.nvidia.com/cuda-zone). The speed of current high-end GPUs is around several TFLOPS (e.g. NVIDIA GeForce GTX 980 at 4.6 TFLOPS), while high-end CPU is normally ∼100 GFLOPS (e.g. Intel Xeon E5-2643 v2 at 168 GFLOPS). Therefore, programs can be potentially accelerated by ∼30 times faster using high-end GPUs in such case.

In addition to GPU, the fast ‘1S2R’ procedure can accelerate Gctf by tens of times more. Since a 2D digital micrograph normally contains thousands of times data points than a 1D curve, an exhaustive search for the defocus and astigmatism in 2D would be incredibly slow. The acceleration by ‘1S2R’ becomes even more significant than GPU when the step size of defocus used for initial search becomes smaller. This is because Gctf only uses the step size for 1D search, which typically takes ∼0.0003 s and is almost ignorable.

The program was further optimized to run as fast as possible by improving the overall strategy (Fig. 2 in (Zhang, 2015)). First, instead of sequentially processing each file (Fig. 2a in (Zhang, 2015)), Gctf tries to process an entire dataset containing hundreds or thousands of micrographs together (Fig. 2b in (Zhang, 2015)). This speeds up the program because a lot of computing resources can be shared among the processing of all the files, the hardware only requires initializing once and sharable parameters, values etc. are also only calculated once (Fig. 2b in (Zhang, 2015)). This concept was not only used in the overall processing of hundreds or thousands of micrographs, but also in lots of the sub-procedures in the entire program. Optimization of file reading strategy can also make the speed of Gctf even faster (Fig. 2c in (Zhang, 2015)).

Apart from the internal acceleration, a convenient script is also provided to help users take advantage of multiple GPU resources (e.g. 10) in their local area network. The scripts can split the whole dataset into several smaller subsets (e.g. 10) and use each GPU to process one subset. It can almost linearly speed up the program on multiple nodes/workstations/PCs using fast parallel file systems.

## 3 Results and discussion

### 3.1 Datasets used for testing Gctf

Table 1 lists a summary of some tested datasets presented in this paper.

### 3.2 Speed test and comparison

The speed of Gctf was tested using different parameters on different devices. In general the speed can be comparable to that of simply reading files. The kernel of CTF fitting only takes ∼0.1 s, using a currently available high-end GPU (e.g. Nvidia GTX 980). In addition to GPU acceleration, which has been shown to accelerate many programs by tens of times (Li et al., 2010, Xu et al., 2010, Zhao and Chu, 2014), Gctf also has significantly improved algorithms and programming strategy (Fig. 3 in (Zhang, 2015)).

Gctf is capable of handling different types of CTF determination and refinement. The limiting factor is mainly the file reading or network speed (Table 2). In a test using dynactin micrographs (Dataset-5, Table 1), the average speed of Gctf can be even accelerated 3 times (from 0.75 s to 0.26 s) simply by using a fast SSD disk. This indicates that the speed limitation is at the file reading step. Changing the pixel size from 1.34 Å (Dataset-5) to 1.70 Å (Dataset-4) or 1.07 Å (Dataset-6) on cryoEM data does not affect the speed at all in this test (with time differences <0.001 s). Similar results were also observed on negative stain datasets (Dataset-2,3).

For movie CTF refinement reading takes 5–30 s but once the movie is read into GPU RAM, processing takes less than 1 s. The limitation for local refinement, however, is mainly the particle number which is approximately linear to the fitting time. It’s much slower than global CTF determination, but very useful to improve the CTF parameters of each particle.

**Fig. 5:**
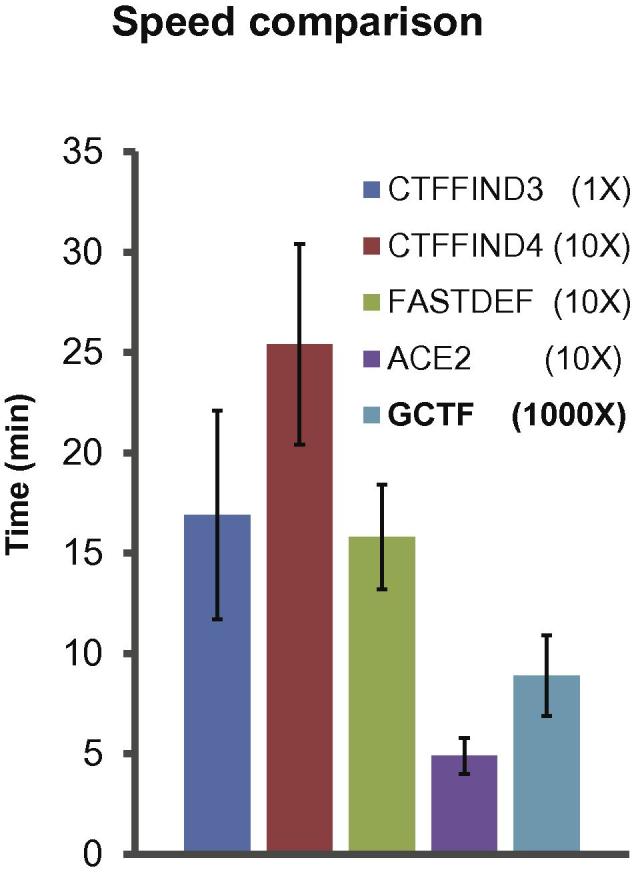
Speed comparison of several popular programs with Gctf. All parameters of each program were set as the default (CTFFIND3/4 called by Relion). Due to the large speed differences among programs, they were tested using different number of micrographs for multiple times: CTFFIND3 on one micrograph, CTFFIND4, FASTDEF, ACE2 on 10 micrographs, while Gctf on 1000 micrographs. Gctf was running on a single GTX 980 GPU and the other programs on Intel Xeon E5-2643 v2 CPU.

Speed comparison was done with CTFFIND3 (Mindell and Grigorieff, 2003), CTFFIND4 (Rohou and Grigorieff, 2015), FASTDEF (Vargas et al., 2013) and ACE2 (Mallick et al., 2005) (Fig. 5). The last three were all claimed to be fast programs, among which ACE2 is the fastest in the current practical test. Gctf using a single GPU card is comparable to one hundred CPU cores by the other three fast programs. Nowadays, people may have alternative choice for doing fast CTF determination using a computer cluster or on a single GPU. However, it might be a better choice to use GPU due to its significantly lower cost.

### 3.3 Accuracy of modified ‘1S2R’ procedure

Micrographs collected under a variety of conditions, including different doses, detectors, magnifications, types of grid, and levels of astigmatism were tested. The plots of 1D cross-correlation function (CCF) each showed a single clear peak (Fig. 3 in (Zhang, 2015)).

One of the major concerns for estimating the averaged defocus using circular averaging is that it might fail due to large astigmatism. However, even in the case of a large astigmatism of ∼10,000 Å ($z_{u}$ = 25,972.84 Å, $z_{v}$ = 15,062.04 Å, θ = 37.22°), Gctf was still able to identify the correct defocus (Dataset-1, Fig. 4 in (Zhang, 2015)). Considering the astigmatism of micrographs under normal cryoEM imaging conditions is less than 1000 Å, and at most 2000 Å, an initial 1D search should work for almost all normal cryoEM micrographs.

The results of global CTF determination were compared with the most popular program CTFFIND3. So far, this program was used to determine the CTF in most near-atomic resolution cryoEM structures (http://www.emdatabank.org/). For a randomly selected subset (123 micrographs) of Dataset-6 (Table 1), the difference between Gctf and CTFFIND3 is ∼40 Å in average (Fig. 5 in (Zhang, 2015)). Both programs could generate 100% essentially correct CTF results using the default parameters (manually checked). FASTED shows a globally smaller defocus (∼400 Å) compared to Gctf or CTFFIND3 with 61.5% essentially correct values using its own default parameters (Fig. 5 in (Zhang, 2015)). ACE2 only failed in three low contrast micrographs but with a bit larger difference (∼600 Å) from Gctf or CTFFIND3 (Fig. 5 in (Zhang, 2015)). It should be noted that all these CTF determination results were generated using the defaults parameters rather than the potential best results from developers. Indeed, the developers can always get better results using their own programs. Also, these results just represent the difference of CTF determination among programs rather the true accuracy.

**Fig. 6:**
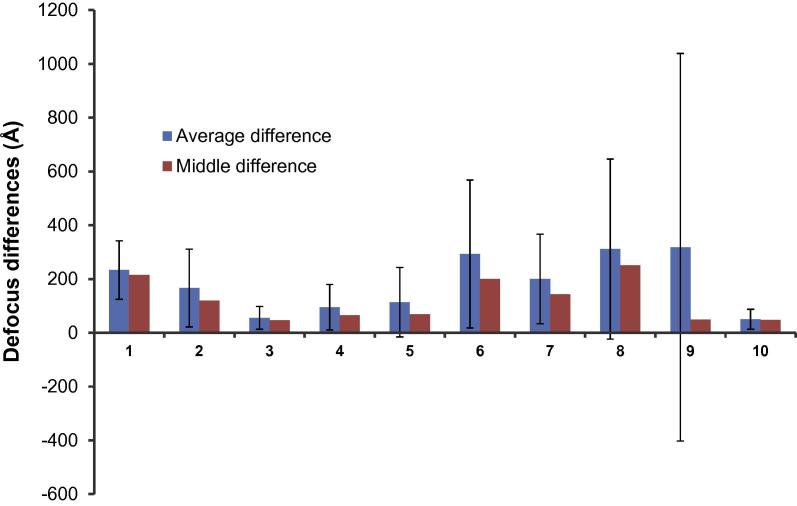
Statistics of the defocus differences between Gctf and CTFFIND3 for the nine benchmark datasets. Number 10 is a dynactin dataset used in Fig. 5 of (Zhang, 2015). Blue columns represent the averaged differences; red columns represent the middle differences, meaning 50% micrographs have the differences lower or higher than this value in each dataset.

Large Gaussian white noise (10 times standard deviation) was added to the micrographs for CTF determination accuracy comparison. All the results except the three low contrast micrographs from both Gctf and CTFFIND3 are still essentially correct and comparable (Fig. 6 in (Zhang, 2015)), in spite of enlarged errors (Gctf 532 ± 96 Å, CTFFIND3 695 ± 125 Å) compared to their averaged defocus of the original micrographs. In contrast, almost none of the CTF determination results were correct from these highly noisy 123 micrographs by FASTDEF or ACE2.

**Fig. 7:**
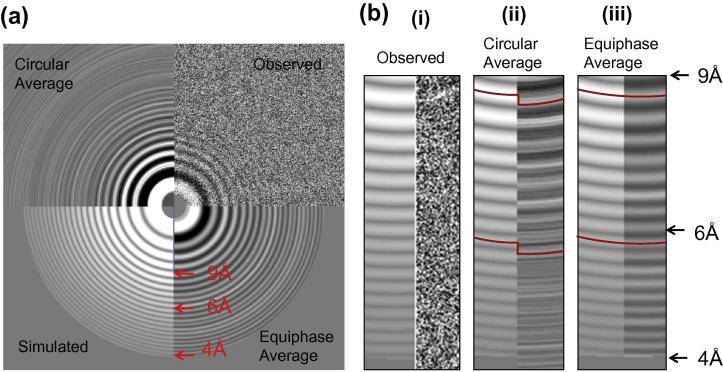
Equiphase average for better diagnosis. (a) Different spectra images of a typical cryoEM micrograph of HAV (Dataset-8) with ∼1800 Å astigmatism; left top: background-subtracted LAS; right top: circular average; left bottom: equiphase average; right bottom: simulated. (b) Enlarged region from 9 Å to 4 Å for detailed comparison of different diagnostic methods. Thong rings on the left sides in all the three images represent the simulated amplitude spectra; the right sides represent the observed spectra visualized in different methods. Obviously, Thong rings from the original spectra (i) are almost invisible at higher than 9 Å even after background reduction. After circular average (ii), the rings become clearer but significantly off the correct peak because of the large astigmatism. Some rings are almost in reverse contrast, indicating the circular average is meaningless at such resolution. The equiphase average (iii) makes all the rings clearly visible up to 4 Å.

During the preparation of this paper, a benchmark study of CTF determination on challenging cases was published (Marabini et al., 2015). Therefore all the datasets were downloaded for testing Gctf. The averaged differences between Gctf and CTFFIND3 (upload 287 by Dr. Grigorieff) are smaller than 400 Å for almost all datasets (Fig. 6). All differences of individual micrographs are also available (Fig. 7 in (Zhang, 2015)). All the raw CTF determination results from Gctf are attached for open comparison (Supplementary Tables S1 and S2 and Supplementary Fig. S1 in (Zhang, 2015)) as well.

The convincing proof of CTF determination accuracy of Gctf came out from several near-atomic resolution maps published recently (Brown et al., 2015, Urnavicius et al., 2015, Zhu et al., 2015) and more to be published soon.

### 3.4 Micrograph evaluation

**Fig. 8:**
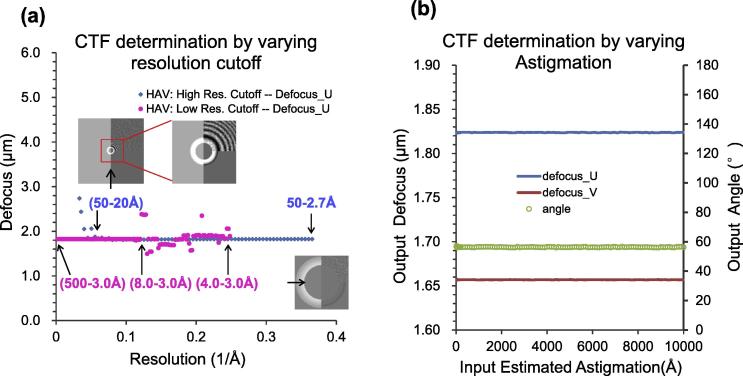
Robustness test of CTF determination using varying resolution cutoff or estimated astigmatism as input. (a) Typical CryoEM micrograph of HAV was selected and systemically examined to test the robustness of Gctf. The blue points represent results by high resolution cutoff and the red points by low resolution cutoff. (b) The input values of astigmatism ranging from 10 Å to 10,000 Å were used as initial estimation for CTF determination. All input values in this range generated almost identical results. Therefore, there is no need for optimizing the input astigmatism in this case. Blue and red lines represent defocus U and V respectively. Green line represents the azimuthal angle.

A summary of CTF determination results by Gctf is presented in Table 2 of (Zhang, 2015) using the described approach (Section 2.10). Several representative Gctf results were shown in Table 2 of (Zhang, 2015). In general, the results on direct electron detectors are much better than those on CCD. In the case of a high quality dynactin dataset on Falcon II detector, 99.7% or 97.9% micrographs are evaluated usable based on 8 Å or 4 Å criterion. The HAV dataset on K2 summit detector shows comparable results. The chaperonin dataset (Zhang et al., 2013) on UltraScan4000 CCD shows worse results, 92.7% are evaluated as usable based on 8 Å criterion and only a quarter is evaluated as usable based on 4 Å criterion. Note that the CTF determination results which were detected to be ‘unusable’ could always be traced to a problem of the micrographs (Fig. 8 in (Zhang, 2015)).

The verification itself might also be affected by high noise at near-atomic resolution. Therefore, manually examination by EPA is highly recommended. A typical micrograph from HAV (Dataset-8) showed clear Thon rings at 4 Å resolution by EPA (Fig. 7b(iii)). Comparable results could not be obtained from original power spectra (Fig. 7b(i)) or circular averaging (Fig. 7b(ii)).

### 3.5 Robustness

Gctf was used to determine the CTFs of different datasets from three examples, dynactin (in Dataset-6), HAV (in Dataset-9) and a pure carbon on Quantifoil grid (in Dataset-7) using variable ranges of four changeable parameters: resolution range, astigmatism, box size and B-factors.

**Fig. 9:**
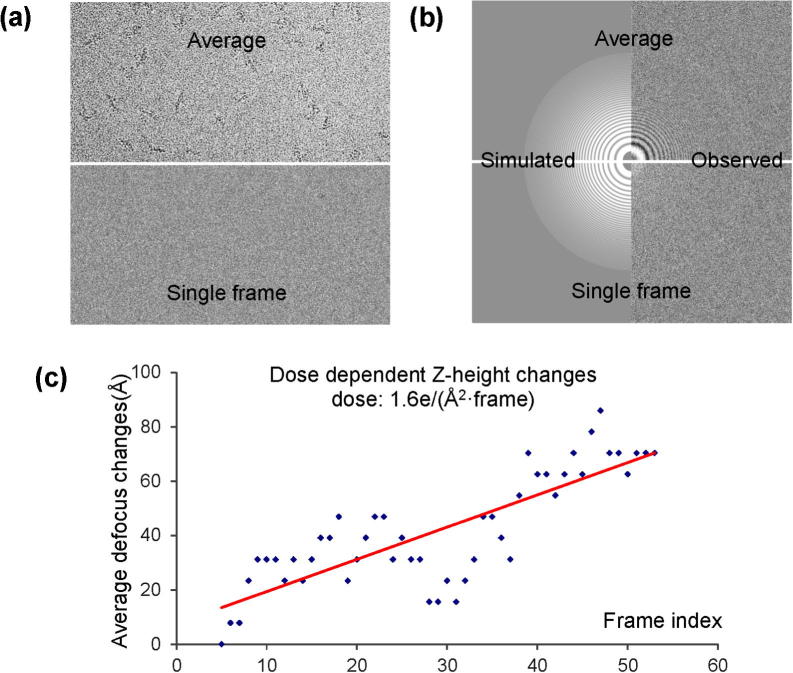
CTF determination of single frames of a movie. (a)Averaged movie (top) and single frame (bottom) of dynactin (Dataset-5). Movie was taken on FEI Titan Krios, Falcon II detector at the dose of 1.6 e/(Å^2^·frame). (b)CTF determination using the averaged movie (top) or a single frame (bottom). For both images, the left is simulated CTF and the right is observed LAS. The determined defocus $\left(z_{u}\text{,}\;z_{v}\text{,}\;\theta_{\mathrm{ast}}\right)$ of the averaged movie is (41,642.58 Å, 41,140.62 Å, 61.67°) and the first frame is (41,711.86 Å, 41,196.36 Å, 52.80°). The difference is (69.28 Å, 55.74 Å, 8.87°). (c) The changes of averaged defocus $(z_{u}+z_{v})/2$ with the accumulation of doses on the micrograph. Slightly different from (b), the CTF determination for each frame was performed by averaging 9 adjacent frames (e.g. 11–19 for frame 15) to enhance the SNR.

Gctf could correctly determine the defocus of an HAV dataset with high resolution cutoffs between Nyquist and 20 Å (e.g. using resolution ranges 500–20 Å, 500–6.0 Å or 500–2.7 Å etc.) or with a low resolution cutoffs between +∞ to ∼8 Å (e.g. 500–3.0 Å, 15–3.0 Å, 8.0–3.0 Å etc.) (Fig. 8a). Movie S1–3 in (Zhang, 2015) are presented to show a clear view of the robustness against a series of resolution cutoffs. Only when the high resolution cutoff was lower than 20 Å (e.g. 500–50 Å, 500–40 Å, 500–30 Å etc.) or the low resolution cutoff was higher than 8 Å (e.g. 6.0–3.0 Å, 5.0–3.0 Å, 4.0–3.0 Å etc.), CTF determination became unstable and the results are not reliable. For dynactin, which was collected on thin carbon film, the resolution cutoff range was more robust (Fig. 9a in (Zhang, 2015)). These results suggest the default resolution range (50–4 Å) for Gctf does not normally need optimization. This is quite helpful for automatic cryoEM data processing.

Gctf can find the correct astigmatism with input values between 10 Å and 10,000 Å for an actual astigmatism of ∼1800 Å (Fig. 8b). This suggests that Gctf can automatically refine the astigmatism of any practical cryoEM micrographs, which usually ranges from 200 Å to 2000 Å, using the default value (1000 Å).

**Fig. 10:**
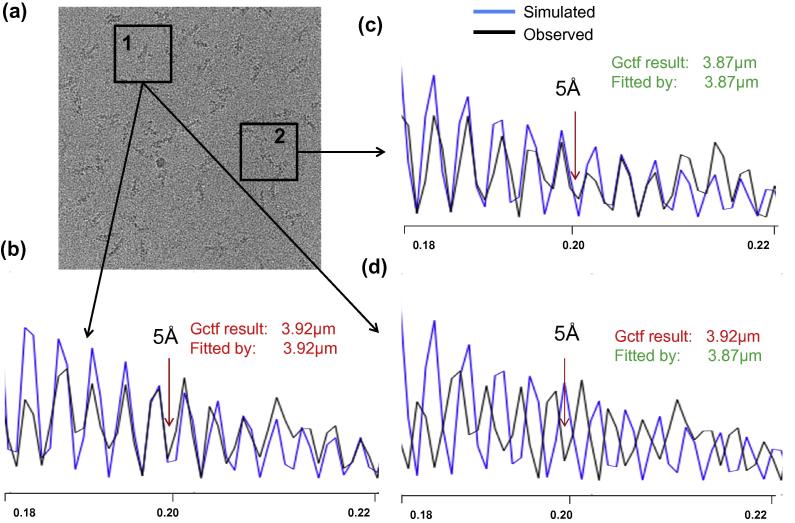
An example showing the importance of local CTF refinement. (a) The raw micrograph of dynactin (Dataset-6). (b) The local defocus for this particle determined by Gctf is 3.92 μm. The red arrow indicates the fitting is almost perfect up to 5 Å. The black curve represents the circularly averaged LAS of particle 1; the blue curve represents the comparison with Gctf determination. (c) Similar to (b) but for particle 2. The defocus of this particle is 3.87 μm. The fitting of CTF is also perfect up to 5 Å. (d) Comparison between the circularly averaged LAS of particle 1 and simulated amplitude spectra using defocus of particle 2. In contrast to (b) or (c), the simulated CTF curve does not fit the observed curve at high resolution, indicating the importance the local refinement for near atomic resolution reconstruction.

The relatively stable range of input Bfactor is around 0–1500 Å^2^ for the HAV Dataset (Fig. 9b and c in (Zhang, 2015)), no need for optimizing the default value (150 Å^2^). In some extreme cases, e.g. very big defocus and astigmatism, CTF determination using a too low Bfactor might not be stable (Fig. 9d in (Zhang, 2015)), while using a too big Bfactor might introduce low-frequency bias because it severely down-weight high frequency. The optimization of Bfactor and resolution range using ‘REBS’ method helps to get better accuracy in such extreme case (Fig. 10 in (Zhang, 2015)).

**Fig. 11:**
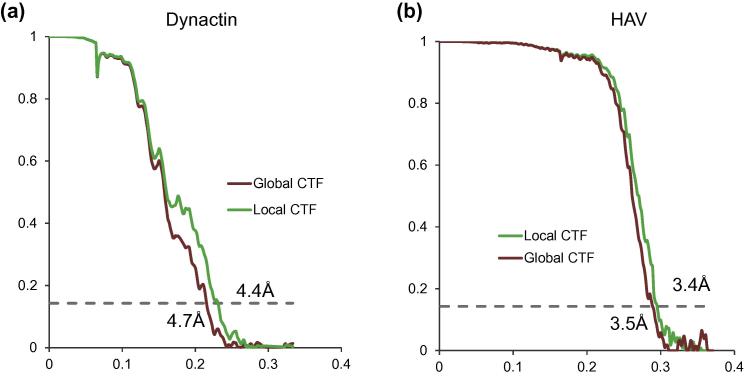
FSC comparison between global and local CTF. (a) Comparison of Dynactin FSC curves with (green) and without (red) doing local defocus refinement. 86,916 particles were used for final reconstruction from Dataset-7. (b) Comparison of FSC curves of HAV with (green) and without (red) doing local defocus refinement. 2025 particles were used for final reconstruction in Dataset-8.

Gctf uses relatively larger box (1024 by default) for better CTF determination because small box affect both accuracy and manual diagnosis (Fig. 11 in (Zhang, 2015)). The big error (∼1100 Å in one case) due to very small box size (128) is not acceptable for near atomic reconstruction (Fig. 1). On the other hand, the box size should not be too big for local CTF refinement. Otherwise, the improvement is not significant. Gctf re-calculates the FFT using new box size 512 for local refinement.

### 3.6 Challenging cases of CTF determination

For easy cases, e.g. micrographs with carbon film at high dose, results from all available CTF determination programs are comparable. The exceptions are cases with large astigmatism since some programs, only determine averaged defocus. The differences among programs become significant for challenging cases. Gctf can accurately determine the CTF for several challenging cases, e.g. low contrast micrographs collected on CCD (Fig. 12a in (Zhang, 2015)), micrographs with very small defocus (e.g. <0.5 μm, Fig. 12b in (Zhang, 2015)), very large defocus (e.g. >7 μm, Fig. 12c in (Zhang, 2015)) or very large astigmatism (e.g. >0.5 μm, Fig. 10 in (Zhang, 2015)) and samples containing ring-like features (e.g. DNA origami), single frames from a movie (Fig. 9a) and so on. Especially, the power of Gctf is demonstrated by its ability to determine the CTF of single frames from a movie with doses of only 1–2 e/Å^2^ (Fig. 9a) (Movie S4 in (Zhang, 2015)). By averaging adjacent frames (e.g. 5–10), the results are accurate enough to detect the changes of *Z*-height (Fig. 9b).

### 3.7 Significant improvement of defocus accuracy by local refinement for each particle

Gctf can estimate the defocus for each particle accurately using the current method described in Section 2.5. One good example, from a high quality dynactin micrograph with a typical defocus variation is shown in Fig. 10. The peaks of the circularly averaged Thon rings are clearly visible for each particle up to 4 Å. A comparison of two representative particles shows that their Thon rings are obviously shifted, almost reversed at higher than 5 Å resolution. A clear comparison between these two particles is shown from Movie S5 in (Zhang, 2015).

The local defocus variation can be much larger than expected from tilt micrographs. The maximum and standard deviation of the averaged local defocus for all micrographs in Dataset-6 were plotted (Fig. 13 in (Zhang, 2015)). The standard deviations of local defocus for over 50% micrographs are actually larger than the theoretical value of a micrograph with 10 degree tilt. The maximum local defocus deviations for about one third micrographs are even larger than the theoretical value of a micrograph with 15 degree tilt. On the other hand, the grid was proved to be flat by the local defocus variation in several regions of completely burned carbon (Fig. 14a in (Zhang, 2015)). In these regions the local defocus variation is smaller than 30 Å which is theoretically equivalent to ∼3 degree tilt. Therefore, the local defocus variation in cryoEM micrographs is not attributed to tilt, but other factors such as uneven ice, carbon support or charging.

In addition to self-consistency verification of global CTF determination, local defocus variation determined by Gctf can also be used to detect abnormal micrographs. Micrographs with very big local defocus deviation (e.g. maximum deviation >1000 Å) were always low-quality (Fig. 14b in (Zhang, 2015)) or partially unusable (Fig. 14c and d in (Zhang, 2015)).

The speed and accuracy of local defocus refinement in Gctf was helpful during the determination of the near-atomic resolution cryoEM structure of dynactin (Urnavicius et al., 2015) (Fig. 15a in (Zhang, 2015)) and HAV (Fig. 15b in (Zhao and Chu, 2014), by courtesy of Wang). Generally, the improvement depends on the magnitude of the defocus variation in the micrographs. Local defocus refinement by Gctf never made the final reconstructions worse in the current test. In one of the best cases for dynactin in a thin carbon layer support, the resolution was improved from 4.7 Å to 4.4 Å (Fig. 11a). In the case of HAV, which was vitrified without carbon substrate and therefore assumed to be less affected by uneven support, local refinement could also improve the resolution from 3.5 Å to 3.4 Å (Fig. 11b).

## 4 Conclusion

Gctf is a convenient, accurate, robust and very fast CTF determination and correction program. GPU acceleration, the fast ‘1S2R’ procedure and optimized programing strategy all together have made it a real-time program. Approaches of self-consistency verification and micrograph quality evaluation have also been proposed for automatic CTF determination and micrograph selection. Approaches for local CTF refinement of each particle in a micrograph or frames in a movie have been proposed to improve the accuracy of CTF determination. Extensive practical tests proved its power to facilitate cryoEM image processing and could improve the final resolution of 3D cryoEM reconstructions in some cases.

## Figures and Tables

**Table 1:** Basic parameters of datasets used to test Gctf in this paper.

Dataset ID	Sample	Type	Microscope	Detectors
1	Empty carbon	Raw grid	Spirit	Orius CCD
2	Dynein	Negative stain	Spirit	Orius CCD
3	Dynactin	Negative stain	Spirit	Orius CCD
4	Dynactin	On thin carbon	Krios	FalconII
5	Dynactin	On thin carbon	Krios	FalconII
6	Dynactin	On thin carbon	Krios	FalconII
7	Dynactin	On thin carbon	Polara	FalconIII
8	HAV	In pure ice	Polara	K2 summit
9	Chaperoin	In pure ice	Krios	UltraScan CCD

ID	apix	kV	Cs	Ac	Dose	Frames	Image size
1	3.3	120	2.0	0.3	20	1	2048 × 2048
2	2.5	120	2.0	0.3	20	1	2048 × 2048
3	3.8	120	2.0	0.3	10	1	2048 × 2048
4	1.70	300	2.7	0.1	51	51	4096 × 4096
5	1.34	300	2.7	0.1	54	34	4096 × 4096
6	1.07	300	2.7	0.1	80	34	4096 × 4096
7	1.38	300	2.2	0.1	54	34	4096 × 4096
8	1.35	300	2.0	0.1	20	20	3712 × 3712
9	0.93	300	2.7	0.04	20	20	4096 × 4096

apix: Pixel size in Ångstrom.

kV: high tension of the micrographs.

Cs: spherical aberration coefficient.

Ac: amplitude contrast used for CTF determination.

Dose: Total dose of all frames, e/Å^2^.

**Table 2:** Typical speed of Gctf for different types of application.^a^

	Dataset-3 (s)	Dataset-9 (s)	Dataset-5 (s)	Dataset-6 (local^b^) (s)	Dataset-5 (movie^c^) (s)
GTX750 Ti, HDD	0.29	0.59	1.14	4.46	4.27
Tesla K40, NFS	0.65	0.76	1.00	3.58	7.88
K5000, NFS	0.68	1.18	1.34	4.31	13.07
GTX980, NFS	0.36	0.60	0.75	3.48	5.60
GTX980, SSD	0.10	0.16	0.26	1.93	2.20

a Time of each micrograph in average.

b 44 particles in total.

c 20 frames, coherent averaging.
